# Cigarette Smoking and Prostate Cancer Mortality in Four US States, 1999–2010

**DOI:** 10.5888/pcd13.150454

**Published:** 2016-04-14

**Authors:** Miranda R Jones, Corinne E. Joshu, Norma Kanarek, Ana Navas-Acien, Kelly A. Richardson, Elizabeth A. Platz

**Affiliations:** Author Affiliations: Corinne E. Joshu, Department of Epidemiology, Johns Hopkins Bloomberg School of Public Health, Baltimore, Maryland, The Sidney Kimmel Comprehensive Cancer Center at Johns Hopkins, Baltimore, Maryland; Norma Kanarek, The Sidney Kimmel Comprehensive Cancer Center at Johns Hopkins, Baltimore, Maryland, Department of Environmental Health Sciences, Johns Hopkins Bloomberg School of Public Health, Baltimore, Maryland; Ana Navas-Acien, Department of Epidemiology and Department of Environmental Health Sciences, Johns Hopkins Bloomberg School of Public Health, Baltimore, Maryland; Kelly A. Richardson, Center for Cancer Prevention and Control, Maryland Department of Health and Mental Hygiene, Baltimore, Maryland; Elizabeth A. Platz, Department of Epidemiology, Johns Hopkins Bloomberg School of Public Health, Baltimore, Maryland, The Sidney Kimmel Comprehensive Cancer Center at Johns Hopkins, Baltimore, Maryland, Department of Urology and the James Buchanan Brady Urological Institute, Johns Hopkins University School of Medicine, Baltimore, Maryland.

## Abstract

**Introduction:**

In the United States, prostate cancer mortality rates have declined in recent decades. Cigarette smoking, a risk factor for prostate cancer death, has also declined. It is unknown whether declines in smoking prevalence produced detectable declines in prostate cancer mortality. We examined state prostate cancer mortality rates in relation to changes in cigarette smoking.

**Methods:**

We studied men aged 35 years or older from California, Kentucky, Maryland, and Utah. Data on state smoking prevalence were obtained from the Behavioral Risk Factor Surveillance System. Mortality rates for prostate cancer and external causes (control condition) were obtained from the Centers for Disease Control and Prevention’s Wide-Ranging Online Data for Epidemiologic Research. The average annual percentage change from 1999 through 2010 was estimated using joinpoint analysis.

**Results:**

From 1999 through 2010, smoking in California declined by 3.5% per year (−4.4% to −2.5%), and prostate cancer mortality rates declined by 2.5% per year (−2.9% to −2.2%). In Kentucky, smoking declined by 3.0% per year (−4.0% to −1.9%) and prostate cancer mortality rates declined by 3.5% per year (−4.3% to −2.7%). In Maryland, smoking declined by 3.0% per year (−7.0% to 1.2%), and prostate cancer mortality rates declined by 3.5% per year (−4.1% to −3.0%).In Utah, smoking declined by 3.5% per year (−5.6% to −1.3%) and prostate cancer mortality rates declined by 2.1% per year (−3.8% to −0.4%). No corresponding patterns were observed for external causes of death.

**Conclusion:**

Declines in prostate cancer mortality rates appear to parallel declines in smoking prevalence at the population level. This study suggests that declines in prostate cancer mortality rates may be a beneficial effect of reduced smoking in the population.

## Introduction

Reductions in the prevalence of smoking subsequent to implementation of tobacco control policies in the United States and other countries are associated with population-level reductions in incidence and mortality from many smoking-associated diseases, including cardiovascular disease, respiratory diseases, and lung cancer (1). In the 2014 Surgeon General’s report, the list of diseases causally associated with smoking was expanded to include prostate cancer (1).

In the United States, prostate cancer mortality rates have declined since the 1990s (2). These declines are due, at least in part, to the combination of prostate specific antigen (PSA)-based prostate cancer screening and better treatment of men diagnosed with prostate cancer and men who progress to metastatic disease (3–6). However, these factors do not entirely explain the decline (3,7). Current cigarette smoking, rather than past or cumulative smoking, is a risk factor for prostate cancer with aggressive pathologic characteristics and increased risk of recurrence and progression among men who have prostate cancer, and for prostate cancer mortality (8). Therefore, the national decline in smoking could have contributed to the decline in prostate cancer mortality rates.

We conducted an ecological study to investigate state-level prostate cancer mortality rates in relation to changes in cigarette smoking among white and black men in 4 US states — California, Kentucky, Maryland, and Utah — during the recent era of declining prostate cancer mortality rates. The 4 states in our study were selected to provide data on a range of population smoking behaviors. As of 2010, Kentucky had the highest prevalence of current smoking among adults in the United States (24.8%), Utah had the lowest prevalence (9.1%), and California (12.1%) and Maryland (15.2%) each had an intermediate prevalence (9). We conducted this study to determine whether population-level tobacco control efforts produced detectable declines in prostate cancer mortality rates in the population.

## Methods

### Study population

We studied men aged 35 years or older, irrespective of race/ethnicity, who resided in 4 US states from 1999 through 2010: California, Kentucky, Maryland, and Utah. We also examined data for non-Hispanic white (white) and non-Hispanic black (black) men aged 35 years or older in the 3 states with available data (California, Kentucky, Maryland). Men aged 35 years or older were selected to capture data on the majority of prostate cancer deaths in the 4 states.

### Prevalence of smoking

Data on the state-specific prevalence of current cigarette smoking for men aged 35 years or older from 1999 through 2010 were obtained from the Behavioral Risk Factor Surveillance System (BRFSS). The BRFSS is a cross-sectional survey conducted by the Centers for Disease Control and Prevention (CDC) and state health departments to obtain state-level estimates of the prevalence of certain health behaviors, diseases, and use of preventive services among noninstitutionalized US adults (10). Response rates for BRFSS 1999–2010 ranged from 35.4% to 58.1% in California, 53.1% to 67.0% in Kentucky, 31.4% to 54.6% in Maryland, and 63.2% to70.6% in Utah (10). To determine current cigarette smoking status, participants were asked, “Have you smoked at least 100 cigarettes in your entire life?” and “Do you now smoke cigarettes every day, some days, or not at all?” Participants were classified as current smokers if they reported smoking at least 100 cigarettes during their lifetime and currently smoked at least some days.

### Mortality outcomes

Annual mortality rates for men aged 35 years or older from 1999 through 2010 were obtained from CDC’s Wide-Ranging Online Data for Epidemiologic Research (CDC WONDER) system (11). CDC WONDER compiles nationwide death certificate data from the National Center for Health Statistics (NCHS). The NCHS captures data on more than 99% of deaths in the United States (12). Prostate cancer deaths were classified according to the *International Classification of Diseases, Tenth Revision *(ICD-10), code C61. As a control condition, we examined mortality rates from external causes (eg, accidents, homicides, suicides; ICD-10 codes V01–Y89), which are not expected to be related to trends in smoking.

### Statistical analyses

For each state, the prevalence of current smoking for men aged 35 years or older was estimated for each year (1999–2010) by using the survey package (version 3.29) in R statistical software (version 3.0.2) (The R Foundation) to account for BRFSS sampling weights. State-specific mortality rates for prostate cancer and external causes for men aged 35 years or older were age-standardized to the 2000 US standard million population and expressed per 100,000 persons. We estimated the average annual percentage change (AAPC) from 1999 through 2010 and the corresponding 95% confidence intervals (CIs) for current smoking, prostate cancer mortality rates, and mortality from external causes by joinpoint analysis (Joinpoint Regression Program, version 4.1.0 [National Cancer Institute] (http://surveillance.cancer.gov/joinpoint/)(13) and compared the observed trends in smoking and mortality outcomes in each state. A maximum of 2 joinpoints, corresponding to 3 segments, were allowed for each analysis. Years for joinpoints were not prespecified. For each state, the locations of the best fitting joinpoints (if any) that indicated a significant change in the slope in that analysis were retained in the final model. Analyses were conducted for all men irrespective of race/ethnicity in California, Kentucky, Maryland, and Utah. We also conducted analyses separately for white and black men in California, Kentucky, and Maryland.

## Results

In 1999, the prevalence of smoking among men aged 35 years or older ranged from 14.8% in Utah to 31.7% in Kentucky (Table 1). By 2010, the smoking prevalence among men had declined in all 4 states (Table 1) (Figure). In California, Kentucky, and Maryland (states with available data on both white and black men), the prevalence of smoking was higher among black men than among white men. In 1999, age-adjusted prostate cancer mortality rates per 100,000 for men aged 35 years or older was 56.1 in California, 65.7 in Kentucky, 68.4 in Maryland, and 64.2 in Utah (Table 1). Similar to the trend observed in smoking, prostate cancer mortality rates also declined in all 4 states by 2010 (Table 1) (Figure).

**Table 1:** Smoking and Mortality Rates for Prostate Cancer and External Causes Among Men Aged 35 Years or Older in 4 States, BRFSS, 1999–2010 and CDC WONDER, 1999–2010

Characteristic	California	Kentucky	Maryland	Utah^a^
**Overall**				
**Number^a^**	9,009,384	1,114,787	1,444,239	553,693
**Current smokers, %^b^**				
1999	19.9	31.7	20.9	14.8
2010	12.8	23.7	15.6	9.4
**Prostate cancer mortality rates**				
Number of deaths^c^	36,318	4,883	6,539	2,242
1999, per 100,000	56.1	65.7	68.4	64.2
2010, per 100,000	41.4	42.3	43.6	51.4
**Death from external causes**				
Number^c^	88,473	16,569	16,405	6,753
1999, per 100,000	89.7	123.6	103.1	115.8
2010, per 100,000	90.0	159.4	97.0	124.2
**Non-Hispanic White Men**				
**Number^a^**	4,603,645	1,006,663	905,567	473,530
**Current smokers, %^b^**				
1999	19.7	31.9	19.0	14.4
2010	11.4	22.6	14.0	8.9
**Prostate cancer mortality rates**				
Number^c^	26,104	4,350	4,156	2,131
1999, per 100,000	57.8	63.0	56.8	63.3
2010, per 100,000	43.6	41.2	33.9	52.5
**Death from external causes**				
Number^c^	57,594	15,575	10,580	6,086
1999, per 100,000	96.6	125.2	92.4	112.8
2010, per 100,000	110.5	168.2	101.5	127.7
**Non-Hispanic Black Men**				
**Number^a^**	550,147	73,278	371,891	5,755
**Current smokers, % b**				
1999	27.3	26.5	30.2	—d
2010	19.5	35.9	21.8	—d
**Prostate cancer mortality rates**				
Number^c^	3,970	502	2,259	—d
1999, per 100,000	125.0	109.5	137.6	—d
2010, per 100,000	86.5	70.5	88.0	—d
**Death from external causes**				
Number^c^	7,430	791	5,139	—d
1999, per 100,000	117.4	113.1	144.7	—d
2010, per 100,000	105.4	80.8	101.5	—d

Abbreviations: BRFSS, Behavioral Risk Factor Surveillance System; CDC WONDER, Centers for Disease Control and Prevention’s Wide-Ranging Online Data for Epidemiologic Research.

a Men aged 35 or older in 2010. The number for overall does not equal the total for whites and blacks because that number includes all men in the state, irrespective of race/ethnicity.

b Values for smoking status are weighted percentages (incorporating sampling weights) for 1999 and 2010.

c Total deaths from select causes from 1999 to 2010

d Insufficient data for current smoking, prostate cancer mortality rates, and mortality from external causes for black men in Utah (≤20 participants for current smoking or ≤20 deaths for mortality outcomes for all years).

**Figure:**
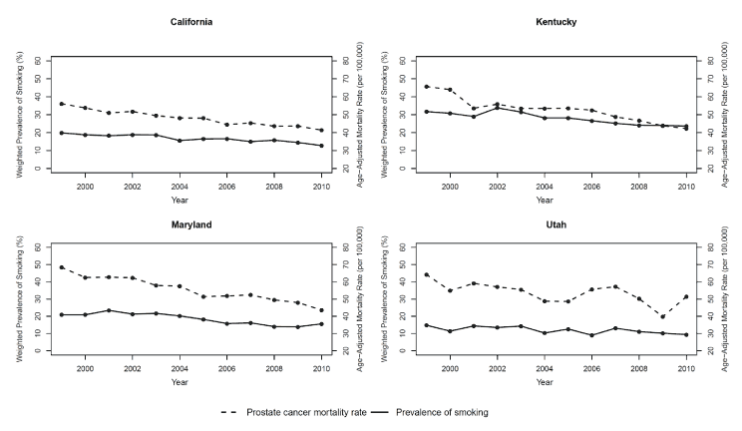
Trends in cigarette smoking and prostate cancer mortality rates among men aged 35 years or older, Behavioral Risk Factor Surveillance System and CDC WONDER, 1999–2010. Solid lines represent trends in the weighted prevalence of cigarette smoking among men aged 35 years or older, and dashed lines represent trends in age-adjusted prostate cancer mortality rates for men aged 35 years or older. Abbreviation: CDC WONDER, Centers for Disease Control and Prevention’s Wide-Ranging Online Data for Epidemiologic Research. **Table:** 

Year	Weighted Prevalence of Cigarette Smoking, %				Age-Adjusted Prostate Cancer Mortality Rate (per 100,000)			
	California	Kentucky	Maryland	Utah	California	Kentucky	Maryland	Utah
1999	19.9	31.7	20.9	14.8	56.1	65.7	68.4	64.2
2000	18.8	30.8	20.9	11.4	53.8	64.0	62.5	54.9
2001	18.3	29.0	23.4	14.4	51.0	53.6	62.7	59.1
2002	18.8	33.8	21.3	13.5	51.8	55.9	62.4	57.1
2003	18.7	31.5	21.7	14.3	49.5	53.5	57.9	55.5
2004	15.6	28.2	20.2	10.3	48.2	53.5	57.5	48.8
2005	16.5	28.2	18.2	12.6	48.1	53.6	51.4	48.6
2006	16.6	26.6	15.8	9.0	44.5	52.5	51.8	55.7
2007	15.0	25.2	16.2	13.1	45.4	48.8	52.4	57.2
2008	15.8	24.1	14.0	11.1	43.6	46.7	49.5	50.2
2009	14.5	24.0	13.9	10.2	43.7	43.8	48.0	39.8
2010	12.8	23.7	15.6	9.4	41.4	42.3	43.6	51.4

In Maryland, which had the highest prostate cancer mortality rates in 1999, the prevalence of smoking among men aged 35 years or older from 1999 through 2010 declined nonsignificantly by 3.0% per year (95% CI, −7.0% to 1.2%), and prostate cancer mortality rates declined by 3.5% per year (95% CI, −4.1% to −3.0%) (Table 2). In Kentucky, which had the second highest prostate cancer mortality rates, the prevalence of smoking declined by 3.0% per year (95% CI, −4.0% to −1.9%), and prostate cancer mortality rates declined by 3.5% per year (95% CI, −4.3% to −2.7%) (Table 2) for the same period. In California, which had the lowest prostate cancer mortality rates in 1999, the prevalence of smoking during this period declined by 3.5% per year (95% CI, −4.4% to −2.5%), and prostate cancer mortality rates declined by 2.5% per year (95% CI, −2.9% to −2.2%). In Utah, the prevalence of smoking declined by 3.5% per year (95% CI, −5.6% to −1.3%) from 1999 through 2010, and mortality rates from prostate cancer declined by 2.1% per year (95% CI, −3.8% to −0.4%). During this period, mortality rates from external causes, which are not expected to be related to population changes in smoking, remained stable in California, Maryland, and Utah and increased significantly in Kentucky (Table 2).

**Table 2:** Average Annual Percentage Change (AAPC)^a^ in Current Smoking and Mortality Rates for Prostate Cancer and External Causes Among Men Aged 35 Years or Older in 4 States, by State and Race, BRFSS, 1999–2010, and CDC WONDER, 1999–2010

Characteristic^b^	California, AAPC (95% CI)	Kentucky, AAPC (95% CI)	Maryland, AAPC (95% CI)	Utah, AAPC (95% CI)^c^
**Overall**				
Current smoker	−3.5 (−4.4 to −2.5)	−3.0 (−4.0 to −1.9)	−3.0 (−7.0 to 1.2)	−3.5 (−5.6 to −1.3)
Prostate cancer	−2.5 (−2.9 to −2.2)	−3.5 (−4.3 to −2.7)	−3.5 (−4.1 to −3.0)	−2.1 (−3.8 to −0.4)
Mortality from external causes	0.8 (−0.3 to 1.9)	1.8 (1.0 to 2.5)	−0.4 (−1.8 to 1.1)	0.5 (−0.1 to 1.2)
**Non-Hispanic white men**				
Current smoker	−4.0 (−5.2 to −2.9)	−3.4 (−4.7 to −2.1)	−3.9 (−5.2 to −2.7)	−3.8 (−5.9 to −1.7)
Prostate cancer mortality rates	−2.4 (−2.8 to −1.9)	−3.4 (−4.4 to −2.4)	−3.7 (−4.6 to −2.9)	−1.8 (−3.5 to 0)
Mortality from external causes	2.1 (1.0 to 3.2)	2.1 (1.3 to 3.0)	1.3 (0.4 to 2.2)	1.1 (0.3 to 1.8)
**Non-Hispanic black men**				
Current smoker	−1.8 (−4.9 to 1.4)	0.7 (−3.5 to 5.0)	−5.0 (−7.4 to −2.6)	—
Prostate cancer mortality rates	−2.7 (−4.1 to −1.2)	−2.8 (−5.9 to 0.4)	−4.0 (−5.4 to −2.6)	—
Mortality from external causes	−0.6 (−4.3 to 3.4)	−2.5 (−4.7 to −0.3)	−2.0 (−3.2 to −0.7)	—

Abbreviations: AAPC, average annual percentage change; BRFSS, Behavioral Risk Factor Surveillance System; CDC WONDER, Centers for Disease Control and Prevention’s Wide-Ranging Online Data for Epidemiologic Research; CI, confidence interval.

a Based on weighted prevalence (for current smoking) or mortality rates age adjusted to the 2000 US standard million population (for prostate cancer mortality rates and mortality from external causes) from 1999 through 2010, analyzed by the Joinpoint Regression Program, version 4.1.0 (National Cancer Institute), allowing up to 2 joinpoints.

b Data on smoking are from BRFSS 1999–2010 (10); data on mortality rates are from CDC WONDER (11).

c Insufficient data for current smoking, prostate cancer mortality rates, and mortality from external causes for black men in Utah (≤20 participants for current smoking or ≤20 deaths for mortality outcomes for all years).

The prevalence of smoking among white men in 1999 ranged from 14.4% in Utah to 31.9% in Kentucky (Table 1). In Kentucky, the prevalence of smoking among white men declined by 3.4% per year (95% CI, −4.7% to −2.1%), and prostate cancer mortality rates similarly declined by 3.4% per year (95% CI, −4.4% to −2.4%) (Table 2) from 1999 through 2010. Among white men in Maryland, both smoking prevalence and prostate cancer mortality rates decreased from 1999 through 2010 (AAPC: −3.9%; 95% CI, −5.2% to −2.7% per year for smoking and −3.7%, 95% CI; −4.6% to −2.9% per year for prostate cancer mortality rates). The declines in prostate cancer mortality rates among white men were smaller in California and Utah compared with Kentucky and Maryland (Table 2). Among white men in California, prostate cancer mortality declined by 2.4% per year (95% CI, −2.8% to −1.9%) and cigarette smoking declined by 4.0% per year (95% CI, −5.2% to −2.9%) from 1999 through 2010. In Utah, prostate cancer mortality rates declined by 1.8% per year (95% CI, −3.5% to 0), and cigarette smoking declined by 3.8% per year (95% CI, −5.9% to −1.7%). During this period, mortality from external causes among white men increased significantly in all states (Table 2).

Although the prevalence of smoking declined significantly among white men in all 4 states, smoking prevalence among black men declined significantly only in Maryland (Table 2). In Maryland, both smoking and prostate cancer mortality rates significantly decreased among black men from 1999 through 2010 (AAPCs: −5.0%; 95% CI, −7.4% to −2.6% per year for smoking and −4.0%; 95% CI, −5.4% to −2.6% per year for prostate cancer mortality rates). Among black men in California, the prevalence of smoking decreased nonsignificantly, by 1.8% per year (95% CI, −4.9% to 1.4%), and mortality from prostate cancer significantly decreased by 2.7% per year (95% CI, −4.1% to −1.2%). In Kentucky, there were no significant changes in the prevalence of smoking or prostate cancer mortality rates for black men (Table 2). In Maryland and Kentucky, black men experienced a significant decline in mortality from external causes, and in California, there was a nonsignificant decrease in mortality from external causes among black men (Table 2).

## Discussion

Using state-level data on smoking behaviors and mortality outcomes, we observed similar declines in the prevalence of smoking and prostate cancer mortality rates among adult men in 4 US states from 1999 through 2010. We also observed greater declines in prostate cancer mortality rates in Kentucky and Maryland during that period, with those states having a higher prevalence of smoking in 1999 compared with California and Utah. We studied concurrent trends rather than lagged trends, because epidemiologic studies indicate that current smoking has important implications for fatal prostate cancer (1). Studies are needed to further evaluate the impact of reductions in smoking in preventing prostate cancer deaths.

Decreases in prostate cancer mortality rates observed at the population level may be due, in part, to decreases in smoking. A 2009 review of prospective epidemiologic studies concluded that current cigarette smoking was associated with a 30% increased risk for fatal prostate cancer compared with nonsmokers (8). When recent smoking was considered (ie, smoking status within 10 years before cancer death), there was a twofold increase in risk for fatal prostate cancer in men who currently smoked or quit within the past 10 years compared with never smokers (8,14,15).

Smoking status at the time of diagnosis is associated with risk for death from prostate cancer (16,17). In the Health Professionals Follow-Up Study (5,366 men diagnosed with prostate cancer from 1986 through 2006), men who reported smoking at the time of diagnosis had increased risk for prostate cancer death (hazard ratio, 1.82; 95% CI, 1.03–3.20) compared with never smokers (17). For prostate cancer mortality, former smokers within 10 years of quitting in the Physicians Health Study (19,705 male physicians recruited from 1982 through 1984) had reduced risk for prostate cancer death compared with current smokers, and after 30 years of cessation, the risk for death from prostate cancer was similar to never smokers (18). At the population level, shifts in prostate cancer mortality rates could be attributed to such changes in smoking behaviors altering the risk for fatal prostate cancer throughout the population.

Although previous studies described the impact of changes in prostate cancer screening or treatment on changes in prostate cancer mortality rates, these changes did not completely explain reductions in prostate cancer mortality rates (3,5). Rates of surgery and radiation therapy to treat localized prostate cancer increased in the United States from the 1980s through the mid-to-late 1990s (3). Information on trends in the use of prostate cancer treatment in the 4 states during this period was unavailable; however, estimates are that changes in treatment (radical prostatectomy, radiation therapy, and androgen deprivation) in the United States could explain 16% to 23% of the decline in prostate cancer mortality rates that occurred from 1991 through 2005 (3). PSA screening began in the United States in the late 1980s and early 1990s and stabilized by the mid-1990s (19). Information on prostate cancer screening was available for men aged 40 years or older in the BRFSS for survey years 2001, 2002, 2004, 2006, 2008, and 2010. Based on these data, the percentage of men who reported being screened for prostate cancer by PSA test or digital-rectal examination remained stable from 2001 through 2010 in Maryland and Kentucky and decreased in California and Utah (AAPCs: −0.8%; 95% CI, −1.8% to 0.1% per year in Maryland; −1.4%; 95% CI, −2.2% to −0.6% per year in California; 1.7%; 95% CI, −0.5% to 4.0% per year in Kentucky; −0.6%; 95% CI, −1.1% to −0.1% per year in Utah). These data suggest that increased PSA screening in these states does not fully explain our findings, although we cannot rule out possible time-delayed effects of earlier (pre-2000) PSA screening and prostate cancer treatment.

We found greater declines in both smoking and prostate cancer mortality rates among black men compared with white men in Maryland, which was the only state in which we observed a significant change in the prevalence of smoking among black men. Compared with white men, black men have a higher incidence of prostate cancer, are more commonly diagnosed with late-stage and high-grade tumors, and have a higher risk for fatal prostate cancer (20). In the United States, black men are also less likely to receive PSA testing (21) or definitive therapy (radical prostatectomy or radiation therapy) than white men (22). Given the lower rates of prostate cancer screening and treatment among black men than white men, our finding of a greater decline in prostate cancer mortality rates among black men than white men in Maryland could not be attributed to racial differences in use of these services.

Despite its low smoking prevalence, Utah had high prostate cancer mortality rates compared with other states in our study. The low prevalence of smoking in Utah is largely due to the state’s religious composition. Members of the Church of Jesus Christ of Latter-Day Saints (LDS), who make up about 70% of Utah’s population (23), practice tobacco abstinence. In Utah in 1996, only 9.2% of LDS men smoked, whereas 24.5% of non-LDS men smoked, a prevalence that exceeded the national average (24).We speculate that the higher rate of prostate cancer mortality in Utah despite an overall low smoking prevalence could be the result of prostate cancer deaths occurring among non-LDS men who are more likely to smoke. However, we do not have information to confirm our speculation.

We used representative data on smoking for men in 4 US states in conjunction with mortality data to examine state-level trends in prostate cancer mortality rates in relation to changes in cigarette use in the population. By examining states with different smoking prevalence and mortality rates we were able to demonstrate that the similarities between prostate cancer mortality rates and smoking trends occurred in different populations. The use of mortality from external causes as a control condition allowed for further comparison of time trends that would not be expected to be influenced by population changes in smoking. We observed no changes in the rates of mortality from external causes over the time period except for an increased trend in Kentucky, which may reflect an increase in the rate of suicide in Kentucky during this time (25).

Our study was an ecological analysis. With this design, we were unable to conclude a causal association, only that the 2 time trends were similar. However, assessing a causal association was not our goal. Instead, we wanted to determine whether declines in the prevalence of cigarette smoking, a risk factor for prostate cancer death, paralleled declines in prostate cancer mortality rates in the population. This study was limited to men in 4 US states, which we selected to provide a range of prostate cancer mortality rates, smoking patterns, racial demographics, and geographic regions; future research is needed to confirm our findings in additional states. The use of state-level data for prostate cancer mortality outcomes does not provide clinical or pathologic information about prostate cancer diagnosis; therefore, we were unable to examine trends in case-fatality rates according to disease characteristics (eg, disease severity at diagnosis). Last, because of our study design we were unable to account for prostate cancer screening and treatment or for possible risk factors (eg, obesity, physical inactivity) and protective factors (eg, statin use) for fatal prostate cancer (26,27). These factors may contribute differences in findings for black men compared with white men in this study.

In the past decade, the prevalence of obesity increased significantly among US adults (28), and levels of physical activity remained stable (29). Thus, the observed decreases in prostate cancer mortality rates occurred despite the upward trend in obesity. Following the 2001 publication of the National Cholesterol Education Program Third Adult Treatment Panel guidelines, which recommended statin use for patients with high levels of low-density lipoprotein (LDL) cholesterol, the percentage of US adults with high levels of LDL cholesterol who reported statin use nearly doubled (from 19.6% in 1999–2000 to 35.9% in 2003–2004) (30). In our study, we did not find a significant change in the trend for prostate cancer mortality rates after 2001 (*P* value for any joinpoints > .05 for all states), and the rates for prostate cancer mortality declined gradually over the period, consistent with a gradual change in risk factors as opposed to the sharp decrease we would expect following the sudden increase of statin use.

From 1999 through 2010, decreasing prostate cancer mortality rates were consistent with a reduction in cigarette smoking at the population level. The association between prostate cancer mortality rates and smoking prevalence was observed in states with various smoking behaviors and prostate cancer mortality rates. Additionally, the lack of change in smoking prevalence among black men in Kentucky was related to unchanged prostate cancer mortality rates in that state. These findings support the need for targeted smoking cessation efforts, which could reduce prostate cancer mortality rates in this population burdened by both higher rates of prostate cancer and an elevated prevalence of cigarette smoking. Finally, these findings support population-wide reductions in smoking as a potential strategy to reduce deaths from prostate cancer.
